# Higher serum trimethylamine-N-oxide levels are associated with increased abdominal aortic calcification in hemodialysis patients

**DOI:** 10.1080/0886022X.2022.2145971

**Published:** 2022-11-16

**Authors:** Lian He, Wenling Yang, Ping Yang, Xianhua Zhang, Aihua Zhang

**Affiliations:** a Department of Nephrology, Peking University Third Hospital, Beijing, China; b Department of Pharmacy, Peking University Third Hospital, Beijing, China; c Department of Nephrology, Xuanwu Hospital Capital Medical University, Beijing, China

**Keywords:** Trimethylamine-N-oxide, abdominal aortic calcification, hemodialysis, vascular calcification, chronic kidney disease, cardiovascular disease

## Abstract

**Introduction:**

Vascular calcification (VC) is high prevalent and predicts cardiovascular mortality in dialysis patients. The mechanisms are not known clearly. Trimethylamine-N-oxide (TMAO), a gut-microbiota derivate metabolite, is also associated with cardiovascular outcomes in hemodialysis (HD) patients. This study aims to evaluate serum TMAO levels and establish their relation to VC in HD patients.

**Methods:**

Serum TMAO concentrations were measured by high-performance liquid chromatography–mass spectrometry. Vascular calcification was evaluated by abdominal aortic calcification (AAC) scores. Taking the AAC score value 5.5 as the cutoff value, the participants were divided into the high AAC score group and the low AAC score group.

**Results:**

A total of 184 HD patients and 39 healthy controls were enrolled in this cross-sectional study. Serum Ln(TMAO) (the natural logarithm of TMAO) concentrations were significantly higher in HD patients than that of control subjects (1.82 ± 0.62 vs. −1.60 ± 0.77, *p* < 0.001). Compared with the group with low AAC scores, the HD patients with high AAC scores showed significantly higher serum Ln(TMAO) levels (2.09 ± 0.55 vs. 1.67 ± 0.54, *p* < 0.001). In the multivariate regression analysis, serum Ln(TMAO), HD vintage, with diabetic mellitus, age and plasma intact parathyroid hormone (iPTH) were independent determinant factors for VC in HD patients.

**Conclusions:**

Higher serum TMAO levels, older age, longer HD vintage, higher plasma iPTH and with diabetes mellitus were independent risk factors for VC in HD patients. The underlying mechanism deserves further investigations and the finding hints at a new target for the treatment of VC.

## Introduction

Vascular calcification (VC) commonly occurs in patients with end-stage renal disease (ESRD) and significantly contributes to the high cardiovascular morbidity and mortality in dialysis patients [1]. VC is an active, highly regulated, and complex biological process. Medial calcification, not intimal calcification, is highly prevalent and special in patients with ESRD, which is characterized by the differentiation of vascular smooth muscle cells (VSMCs) into osteoblast-like cells [2]. However, the pathogenesis of VC in chronic kidney disease (CKD) state is very complicated and is not fully elucidated.

Presently, it was reported that patients with CKD showed intestinal dysbiosis, an alteration of the gut micro-organism composition and function [3]. Gut dysbiosis was linked to cardiovascular diseases (CVDs), such as hypertension, heart failure in general population [4,5]. Trimethylamine-N-oxide (TMAO) is a small, organic, gut microbiota-derived metabolite, which is emerging as a new and potentially important risk factor for atherosclerosis and CVDs in the general population. To date, there are several studies demonstrating that markedly elevating serum TMAO levels were associated with cardiovascular outcomes in patients receiving dialysis [6–8]. In a United States multicenter study enrolling over 1000 hemodialysis (HD) patients, TMAO concentrations were associated with cardiovascular events [6]. Also in a few relatively smaller studies in China, the positive associations of TMAO levels with cardiovascular, all-cause mortality [8] and the incidence of hospitalization events [9] were reported in HD patients. Although previous studies have observed the relation between serum TMAO and CVD events in dialysis patients, the role of TMAO in the pathogenesis of CVD are still not clear completely. Recently, Lin et al. found that TMAO affected the balance of bone metabolism and accelerated osteoporosis *in vitro* [10]. VC shares a similar etiology with bone disease in CKD [11].

Thus, we speculate that TMAO may, through increasing VC, contribute to CVD in ESRD patients. In this study, we will investigate the association between serum TMAO and VC in HD patients. Until now, few similar clinical studies have been reported. Based on our study, we want to acquire some clues for the probable therapeutic pathways for VC, such as applying probiotics, in patients with uremia in the future.

## Materials and methods

### Subjects

**Figure 1.**
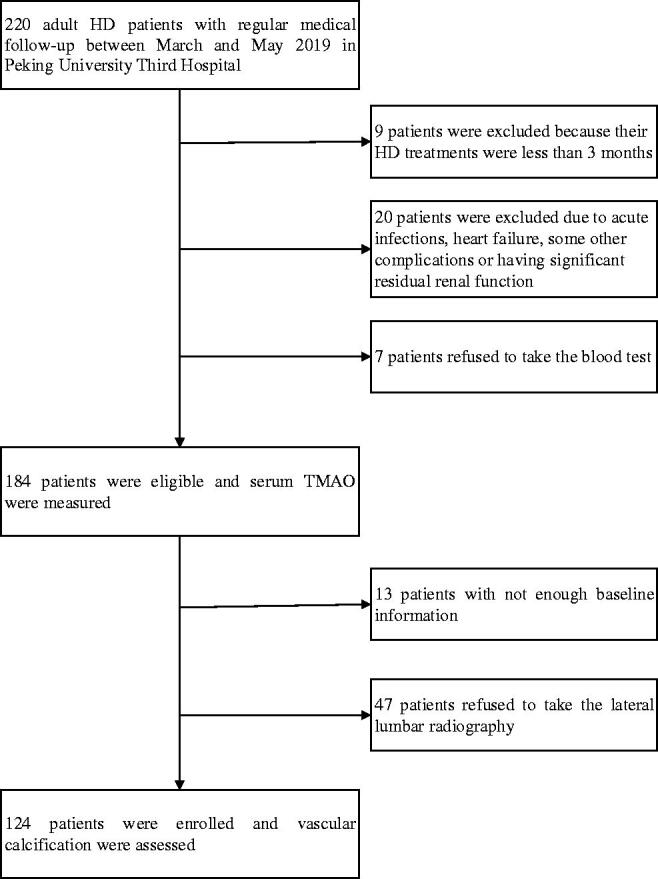
Flowchart of the study. HD: hemodialysis; TMAO: trimethylamine-N-oxide.

This cross-sectional study screened 220 HD patients and enrolled 184 maintenance HD patients and 39 healthy controls in the Peking University Third Hospital between March and May 2019. Patients were eligible for inclusion if they (1) were aged >18 years old and (2) had been on HD for at least 3 months. The exclusion criteria included (1) in the acute phase of infections, heart failure, or some other complications, (2) with significant residual kidney function (urinary urea clearance >1.5 mL/min, according to previous studies [6,12]), and (3) refusal to participate in the study. A flowchart of patient recruitment for the study is shown in Figure 1.

The study complied with principles laid down by Declaration of Helsinki and was approved by the Ethics Committee of the Peking University Third Hospital (IRB00006761-M2019003). Written informed consent was provided by all participants. Authors had not access to information that could identify individual participants during or after data collection.

### Clinical and biochemical data collection

Data collected included patient demographics such as age, gender, underlying cause of ESRD and HD vintage. Laboratory data collected at baseline included hemoglobin, albumin, corrected calcium, phosphorus, intact parathyroid hormone (iPTH) level, ultrasensitive C-reactive protein (usCRP), and lipid profile. Fractional urea clearance (*Kt*/*V* urea) was calculated by the Daugirdas formula.

### Measurement of TMAO

Blood samples were collected in vacuum tubes without anticoagulant after fasting for at least 8 h and pre dialysis. Following centrifugation, serum was collected and stored at –80 °C until analysis. Serum TMAO levels were determined using high-performance liquid chromatography–mass spectrometry (HPLC–MS) [13].

### Assessment of vascular calcification

Vascular calcification was imaged within 2 weeks of enrollment: abdominal aortic calcification (AAC) by lateral lumbar radiography with Kauppila’s scoring [14]. According to the previous studies [15,16], the point with AAC score of 5.5 was reported to determine the severity of coronary artery disease in the dialysis population. Thus, taking the AAC score value 5.5 as the cutoff value, patients were allocated to the high AAC score group if they had an AAC score of ≥5.5 (moderate or heavy calcification) and the low AAC score group if they had an AAC score of <5.5 (no or minor calcification).

### Statistical analysis

Results are expressed as proportions (percentages) for categorical variables, mean ± standard deviation for continuous normally distributed variables, and median with interquartile ranges for continuous non-normally distributed variables. Student’s *t*-test was used to compare differences between the two groups for normally distributed data, while the Mann–Whitney *U*-test was used for non-normal data. Categorical data were compared using the Chi-square test. Correlations were expressed as Pearson’s correlation coefficients for two normally distributed variables, and Spearman’s rank correlations were used for non-normally distributed variables. Serum TMAO, the non-normally distributed data, were changed to Ln(TMAO) (the natural logarithm of TMAO) for Pearson’s correlated analysis and Student’s *t*-test. The multivariate linear regression model (using backward conditional method) was employed to select variables independently related to serum Ln(TMAO). Binary logistic regression analysis (using backward conditional method) was employed to select variables independently related to VC, with all factors related to VC included in the model. Receiver operating characteristic (ROC) curve analysis found the best cutoff value of Ln(TMAO) for VC (a high AAC score ≥ 5.5) prediction. All analyses were two-tailed, and a *p* < 0.05 was considered statistically significant. SPSS Software, version 16.0 (SPSS, Inc., Chicago, IL) was used for all statistical analyses.

## Results

### Baseline characteristics of subjects

One hundred and eighty-four HD patients were enrolled according to the inclusion and exclusion criteria. The primary renal diseases were mainly diabetes (50 patients, 27.2%) and chronic glomerulonephritis (61 patients, 33.1%). Other demographic and clinical characteristics of HD patients and 39 healthy controls are shown in Table 1. In HD patients, the serum TMAO concentration was 5.80 (3.96, 9.46) µg/mL, whereas in controls it was 0.18 (0.11, 0.32) µg/mL. Serum Ln(TMAO) were significantly higher in HD patients than that of control subjects (1.82 ± 0.62 vs. −1.60 ± 0.77, *p* < 0.001). Since age or gender were not matched well between the controls and the patients, in order to adjust age and gender, a multivariate linear regression analysis for Ln(TMAO) was done in all participants (controls and HD patients). Age, gender, different group number (HD patients = 1, controls = 2), and other confounders in Table 1 were included in the analysis. Consequently, different group was independently related to Ln(TMAO) (*p* < 0.001). It meant that the difference of serum Ln(TMAO) level between HD patients and controls still existed after adjusting age, gender, and some other confounders.

**Table 1.** Comparison of clinical parameters between hemodialysis patients and normal controls.

Characteristics	Hemodialysis patients (*n* = 184)	Normal controls (*n* = 39)	*t*/*κ*2/*Z* value	*p* Value
Age (years)	60.1 ± 14.8	44.8 ± 12.6	6.002	<0.001
Gender (male, *n*, %)	123,66.8	15,38.5	10.993	0.001
Dialysis vintage (months)	56.0 (32.6–105.4)			
Hemoglobin (g/L)	108.2 ± 11.6	144.0 ± 15.1	–13.930	<0.001
Albumin (g/L)	40.6 ± 3.4	44.8 ± 2.4	–7.115	<0.001
usCRP (mg/L)	2.81 (0.87,6.61)	0.72 (0.17–2.11)	–3.515	<0.001
Serum urea (mmol/L)	26.1 ± 5.6	4.7 ± 1.2	46.911	<0.001
Serum creatinine (µmol/L)	957.9 ± 269.3	72.9 ± 12.1	44.369	<0.001
Serum TMAO (µg/mL)	5.80 (3.96, 9.46)	0.18 (0.11, 0.32)	–9.782	<0.001
Ln(TMAO)	1.82 ± 0.62	–1.60 ± 0.77	29.835	<0.001

usCRP: ultrasensitive C-reactive protein; Ln(TMAO): the natural logarithm of trimethylamine-N-oxide.

### Correlation analysis of serum Ln(TMAO) with other parameters in HD patients

Bivariate correlation analysis revealed that serum Ln(TMAO) was positively correlated with dialysis vintage (Spearman’s *r* = 0.160, *p* = 0.030), serum urea (Pearson’s *r* = 0.176, *p* = 0.017), triglycerides (TGs) (Spearman’s *r* = 0.272, *p* < 0.001), and diabetic mellitus (Spearman’s *r* = 0.168, *p* = 0.023), while negatively correlated with serum high-density lipoprotein cholesterol (HDL-C) (Pearson’s *r*=−0.164, *p* = 0.027). Furthermore, circulating Ln(TMAO) was also positively correlated with AAC scores (Spearman’s *r* = 0.286, *p* = 0.002) (see Table 2).

**Table 2.** Correlation analysis of serum Ln(TMAO) with other parameters in HD patients (*n* = 184).

Variables	*r* [Ln(TMAO)]	*p* Value
Age (years)	0.092	0.214
Gender (male = 1, female = 2)	–0.029	0.698
Diabetic mellitus (no = 0, yes = 1)	0.168	0.023
Dialysis vintage (months)	0.160	0.030
Hemoglobin (g/L)	0.095	0.199
Serum albumin (g/L)	0.116	0.116
Serum urea (mmol/L)	0.176	0.017
Serum creatinine (µmol/L)	0.079	0.285
Serum corrected calcium (mmol/L)	0.072	0.329
Serum phosphorus (mmol/L)	0.087	0.239
iPTH (pg/mL)	0.048	0.523
Serum usCRP (mg/L)	0.102	0.176
Serum triglycerides (mmol/L)	0.272	<0.001
Serum LDL-C (mmol/L)	0.084	0.260
Serum HDL-C (mmol/L)	–0.164	0.027
*Kt*/*V* urea	0.107	0.156
AAC score	0.286	0.002

Ln(TMAO): the natural logarithm of trimethylamine-N-oxide; HD: hemodialysis; iPTH: intact parathyroid hormone; usCRP: ultrasensitive C-reactive protein; LDL-C: low-density lipoprotein cholesterol; HDL-C: high-density lipoprotein cholesterol; *Kt*/*V* urea: fractional urea clearance; AAC: abdominal aortic calcification.

In order to remove the impact of confounding factors on Ln(TMAO), multivariate linear regression analysis was done. In the analysis, all the factors related or having the tendency related to Ln(TMAO) in Table 2 were included as candidate variables, such as dialysis vintage, diabetes mellitus, serum urea, serum TG, serum HDL-C, serum usCRP, and serum albumin. At the end, the multivariate regression analysis (backward method) showed that diabetes mellitus, serum urea, and dialysis vintage were independent variables related to serum Ln(TMAO) levels in HD patients (see Table 3).

**Table 3.** Independent influencing factors of serum Ln(TMAO) by multivariate linear regression analysis in HD patients^a^.

Variables	First step		Last step	
	*B*	*p* Value	*B*	*p* Value
Constant	0.771	0.224	1.041	<0.001
Dialysis vintage (months)	0.003	0.002	0.003	0.001
Diabetic mellitus (no = 0, yes = 1)	0.285	0.005	0.309	0.002
Serum urea (mmol/L)	0.017	0.047	0.019	0.018
Serum triglycerides (mmol/L)	0.027	0.414	Excluded	
Serum HDL-C (mmol/L)	–0.139	0.355	Excluded	
Serum usCRP(mg/L)	0.001	0.801	Excluded	
Serum albumin (g/L)	0.010	0.485	Excluded	

Ln(TMAO): the natural logarithm of trimethylamine-N-oxide; HD: hemodialysis; HDL-C: high-density lipoprotein cholesterol; usCRP: ultrasensitive C-reactive protein; *B*: partial regression coefficient.

^a^ All factors correlated or having the tendency correlated to serum Ln(TMAO) in Table 2, including dialysis vintage, diabetic mellitus, serum urea, triglycerides, HDL-C, usCRP, and serum albumin, were included in the multivariate linear regression model, using backward conditional methods for analysis.

### Comparison of serum Ln(TMAO) levels and other established parameters between the high and the low AAC scores group

**Figure 2.**
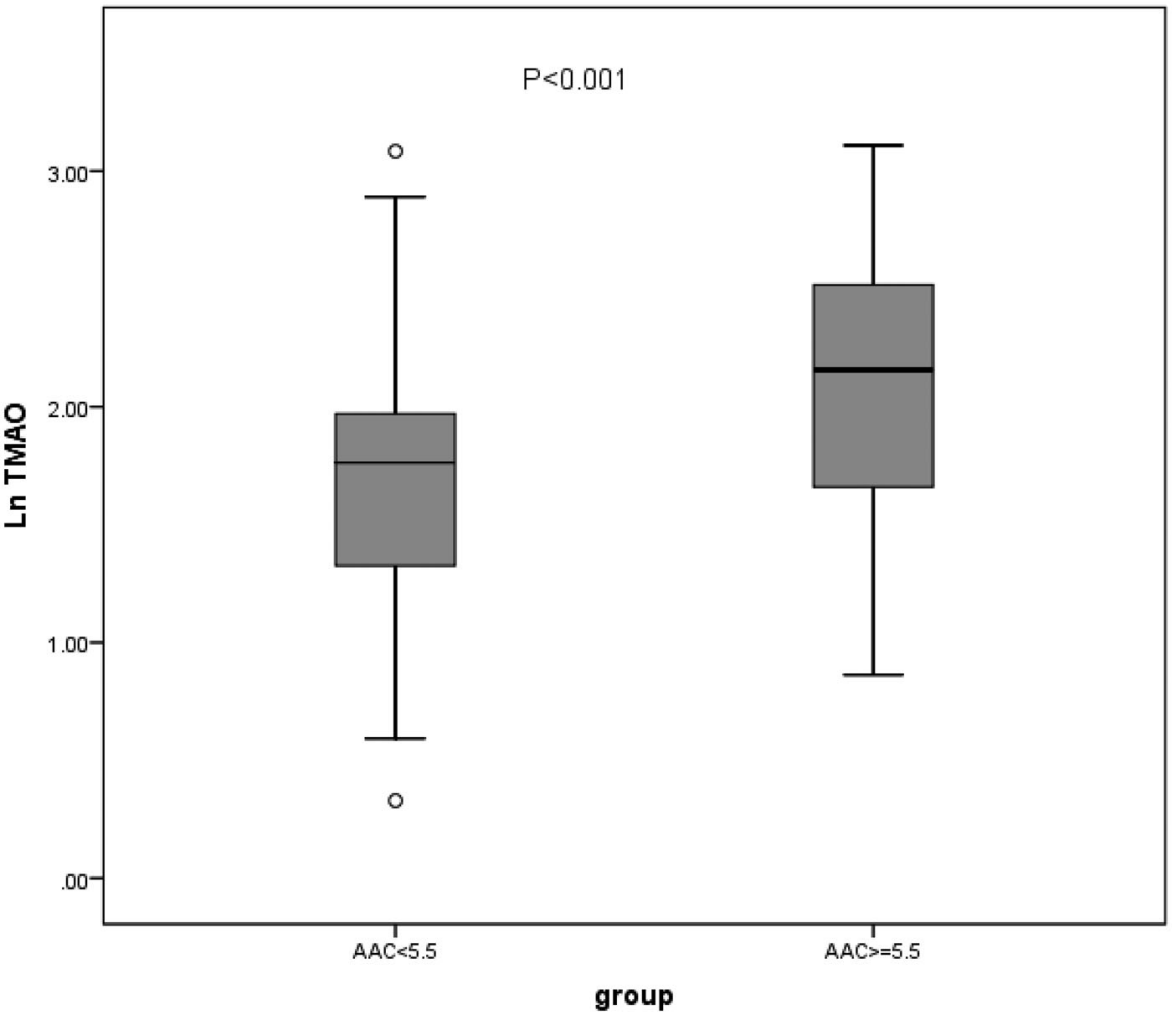
Comparison of serum Ln(TMAO) between the high AAC score group and the low AAC score group. Ln(TMAO): the natural logarithm of trimethylamine-N-oxide; AAC: abdominal aortic calcification.

Compared with the low AAC score group, the HD patients with high AAC score showed significantly higher serum Ln(TMAO) levels (2.09 ± 0.55 vs. 1.67 ± 0.54, *p* < 0.001) (see Table 4 and Figure 2). The HD patients with high AAC score had lower serum creatinine levels than the low AAC score group had (*p* = 0.002). Meanwhile, there were significantly older age (*p* < 0.001), longer HD vintage (*p* < 0.001), higher plasma iPTH levels (*p* = 0.005), and more diabetes mellitus (*p* = 0.029) in HD patients with high AAC score than those in the low AAC score group. We can see higher serum usCRP level in the high AAC score group, but it was not statistically significant (*p* = 0.076). Additionally, there were no differences in serum calcium, phosphate, lipids levels, and gender between the two groups (all *p* > 0.05) (see Table 4).

**Table 4.** Comparison of serum Ln(TMAO) and other parameters between the high AAC score group and the low AAC score group.

Characteristics	AAC score < 5.5 (*n* = 67)	AAC score ≥ 5.5 (*n* = 57)	*p* Value
Age (years)	53.3 ± 14.2	66.0 ± 11.3	<0.001
Gender, male, *n* (%)	44 (65.7)	35 (61.4)	0.622
Dialysis vintage (months)	53.9 (22.4, 74.3)	75.2 (50.7, 142.1)	<0.001
Diabetes mellitus, *n* (%)	19 (28.4)	27 (47.4)	0.029
Hemoglobin (g/L)	108.6 ± 9.9	109.3 ± 11.8	0.722
Serum creatinine (µmol/L)	1044.9 ± 311.8	897.5 ± 193.9	0.002
*Kt*/*V* urea (per week)	1.28 ± 0.25	1.34 ± 0.17	0.125
Serum albumin (g/L)	41.1 ± 3.3	40.5 ± 2.9	0.351
Serum corrected calcium (mmol/L)	2.26 ± 0.18	2.28 ± 0.21	0.655
Serum phosphate (mmol/L)	1.80 ± 0.52	1.77 ± 0.48	0.776
Serum usCRP (mg/L)	2.07 (0.58, 6.56)	3.39 (1.37, 8.12)	0.076
Plasma iPTH (pg/mL)	229.1(112.5, 443.5)	368.0(212.2, 600.1)	0.005
Serum CO2CP (mmol/L)	19.7 ± 3.0	19.6 ± 2.2	0.844
Serum LDL-C (mmol/L)	2.02 ± 0.63	1.96 ± 0.67	0.596
Serum HDL-C (mmol/L)	1.00 ± 0.34	0.96 ± 0.35	0.530
Serum triglycerides (mmol/L)	1.64 (0.92, 2.50)	1.98 (1.18, 2.89)	0.123
Serum Ln(TMAO)	1.67 ± 0.54	2.09 ± 0.55	<0.001
AAC (score)	2 (0, 3)	9 (6.5, 13)	<0.001

Ln(TMAO): the natural logarithm of trimethylamine-N-oxide; AAC: abdominal aortic calcification; *Kt*/*V* urea: fractional urea clearance; usCRP: ultrasensitive C-reactive protein; iPTH: intact parathyroid hormone; CO2CP: carbon dioxide combining power; LDL-C: low-density lipoprotein cholesterol; HDL-C: high-density lipoprotein cholesterol.

### Independent risk factors of vascular calcification by binary logistic analysis in HD patients

In order to remove the impact of confounding factors on VC, a multivariate logistic regression model was performed to find the independent risk factors of VC in HD patients. The variables which significantly correlated to a high AAC score of ≥5.5 in univariate analysis, including age, dialysis vintage, diabetic mellitus, serum creatinine, plasma iPTH, serum Ln(TMAO), and usCRP which had a tendency of significant association (shown in Table 4) entered the analysis as candidate variables. Finally, age, dialysis vintage, diabetic mellitus, plasma iPTH, and serum Ln(TMAO) were independently associated with a high AAC score in HD patients (all *p* < 0.05) (see Table 5).

**Table 5.** Independent related factors of a high AAC score ≥ 5.5 by multivariate binary logistic analysis in HD patients^a^.

Variables	First step			Last step		
	*B*	*p* Value	95% CI for Exp(*B*)/OR	*B*	*p* Value	95% CI for Exp(*B*)/OR
Age (years)	0.093	0.002	1.035–1.164	0.096	<0.001	1.049–1.155
Dialysis vintage (months)	0.014	0.010	1.003–1.025	0.014	0.009	1.004–1.025
DM (no = 0, yes = 1, 1 vs. 0)	1.255	0.044	1.033–11.910	1.318	0.026	1.172–11.908
Serum creatinine (µmol/L)	–0.0003	0.818	0.997–1.003	excluded		
Serum usCRP (mg/L)	–0.013	0.692	0.926–1.052	excluded		
Plasma iPTH (pg/mL)	0.002	0.008	1.001–1.004	0.002	0.008	1.001–1.004
Serum Ln(TMAO)	1.261	0.014	1.296–9.604	1.263	0.011	1.328–9.406
Constant	–10.200	0.001		–10.765	<0.001	

AAC: abdominal aortic calcification; HD: hemodialysis; DM: diabetes mellitus; usCRP: ultrasensitive C-reactive protein; iPTH: intact parathyroid hormone; Ln(TMAO): the natural logarithm of trimethylamine-N-oxide; *B*: partial regression coefficient; Exp(B)/OR: odds ratio; CI: confidence interval.

^a^ All factors correlated to vascular calcification in Table 4, including age, dialysis vintage, DM, serum creatinine, usCRP, iPTH, and Ln(TMAO), were included in the multivariate logistic regression model, using backward conditional methods for analysis.

### ROC curve analysis of serum Ln(TMAO) level and a high AAC score ≥ 5.5 in hemodialysis patients

**Figure 3.**
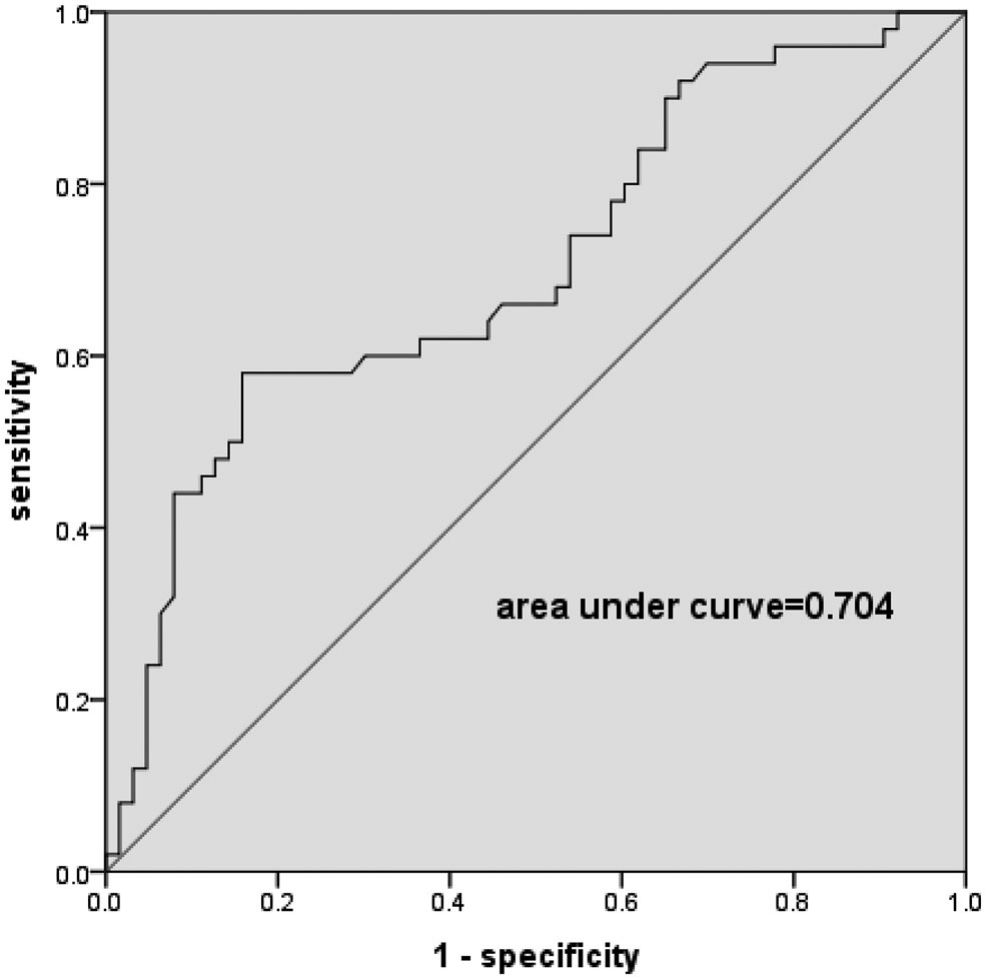
Receiver operating characteristic (ROC) analysis of serum Ln(TMAO) and the risk of a high AAC score ≥ 5.5 in hemodialysis patients. ROC study showed an area under curve of 0.704 (*p* < 0.001). A serum Ln(TMAO) greater than 2.07 was associated with a high AAC score ≥ 5.5 occurrence with a sensitivity of 0.580 and a specificity of 0.841. Ln(TMAO): the natural logarithm of trimethylamine-N-oxide; AAC: abdominal aortic calcification.

ROC analysis identified the best serum Ln(TMAO) cutoff value for a high AAC score ≥5.5 prediction. It showed that a serum Ln(TMAO) of 2.07 was associated with a high AAC score ≥5.5 with a sensitivity of 0.580 and a specificity of 0.841. The area under curve (AUC) was 0.704 (*p* < 0.001) (see Figure 3). The Ln(TMAO) value of 2.07 equals the TMAO value of 7.92.

## Discussion

In the current study, our results showed that serum TMAO levels, one of the gut microbiota-derived metabolite, were significantly higher in maintenance HD patients than that in controls. Importantly, we demonstrated for the first time that serum TMAO concentrations were notably higher in the high AAC score group than that in the low AAC score group in HD patients. Moreover, higher serum TMAO was an independent risk factor for VC in HD patients. Thus, these findings supported our hypothesis that through VC, TMAO may contribute to CVD in ESRD patients.

We observed that the circulating TMAO concentrations in HD patients were about 30 times higher than that in healthy controls. It was similar to the data from Shafi et al.’s, Zhang et al.’s, and Tang et al.’s studies [6,8,17]. The mechanisms of a significant increase in circulating TMAO in late-stage CKD could be multifactorial. TMAO is derived primarily from metabolization of trimethylamine (TMA)-containing substrates, such as dietary carnitine, choline, and betaine, through the action of gut microbiota [18]. HD patients had to restrict their diets, take medicine and thus the gut microbiota would alter due to the changed eating habits, all kinds of drugs and uremia state [3]. In addition, TMAO is a low-molecular-weight (75 Da) metabolite, which is excreted in the urine [17]. It also can be removed efficiently like urea during a single HD session. However, it accumulates between HD sessions in HD patients [19]. Hence, both impaired renal function and dysbiosis of the gut microbiota contribute to the elevated serum TMAO levels in HD patients [20]. In this study, the HD patients with significant residual kidney function were excluded to minimize the effect of residual kidney function.

The most important and interesting thing was that we found higher serum TMAO was an independent risk factor for VC in HD patients. It was independent of serum creatinine and dialysis adequacy (*Kt*/*V*). As far as we know, it is the first clinical study about the relation between TMAO and AAC in HD patients. Vascular calcification is a special risk factor for high cardiovascular mortality and morbidity of CKD cases [1]. The medial VC promoted by hyperphosphatemia is the key process in CKD patients [2]. TMAO, a gut microbiota-derived metabolite, was reported to relate to adverse cardiovascular events in uremia patients [6]. Our study’s results demonstrated a clinical link between VC and TMAO in HD patients. Further, the underlying mechanisms need to be elucidated. Recently, Zhang et al. [21] revealed that TMAO promoted calcium-induced osteogenic differentiation of VSMCs and also exacerbated VC in rats with CKD, which were involved activation of NLRP3 (nucleotide-binding domain, leucine-rich containing family, pyrin domain-containing-3) inflammasome and nuclear factor κB (NF-κB) signaling pathways. In fact, apart from VSMCs trans-differentiation, VSMCs apoptosis, autophagy, oxidative stress, chronic inflammation, endothelial dysfunction, loss of mineralization inhibitors, release of calcifying extracellular vesicles and multiple signaling pathways are all recognized to drive the VC process now [2,22]. The mechanisms of TMAO promoting VC in CKD deserve to be further studied. Based on these results, changing gut flora and decreasing the TMAO levels maybe a potential therapy for alleviating VC in dialysis patients. However, another clinical study showed that TMAO concentration was not associated with coronary artery calcium incidence in non-CKD young healthy adults [23]. We thought the young healthy adults in that study were very different from HD patients in our study and the results were not comparable between each other. In this study, in consideration of the confounding factor of age, multivariate regression analysis has been done in finding the independent risk factor of VC in HD patients and TMAO was still the risk factor of VC independently.

In addition, by ROC curve analysis, we found serum Ln(TMAO) greater than 2.07 or serum TMAO greater than 7.92 predicted VC (AAC score ≥ 5.5) existence. However, the cutoff value 7.92 of TMAO related to VC needs to be verified in other HD cohort in the future. Previous studies [15,16] reported that the cutoff value 5.5 of AAC score can predict CVD in ESRD patients. Hence in this study, VC group was divided according to the AAC score 5.5. It is also worth further study to clarify whether the cutoff value 7.92 of TMAO can predict the occurrence of CVD in HD patients.

The traditional risk factors of VC in dialysis patients include old age, prolonged dialysis vintage, diabetic mellitus, hyperphosphatemia, high iPTH level, and inflammation [24]. Consistently, our results showed that older age, longer dialysis vintage, with diabetic mellitus and higher iPTH were also independent related factors of AAC in HD patients. However, no significant correlations between VC and serum phosphorus, inflammation were observed in our study, possibly due to the relatively small sample size, the generally well-controlled serum phosphorus or inflammation, and the single serum phosphorus and inflammation biomarker data used in our study.

In general population, diabetes mellitus was associated with higher plasma TMAO levels [25]. Obeid et al. [26] proposed that the elevated plasma TMAO concentrations were likely to reflect a specific metabolic pattern characterized by low high-density lipoprotein (HDL) and phospholipids. Evidence suggested that TMAO played a key regulatory role in glucose and lipid metabolism [25,27]. In HD patients, similar studies were rare. Shafi et al.’s results showed diabetes was associated with higher TMAO concentrations in HD populations [6]. Our present study also revealed the same results and diabetes was an independent factor related to the serum TMAO levels in HD patients. In addition, we found higher serum TGs and lower HDL-C levels were associated with higher TMAO levels in HD patients, although in multivariate linear regression analysis they were not independent influencing factors of TMAO. Hence even in uremia, TMAO still appeared to regulate glucose and lipid homeostasis, which could also act as an important determinant for atherosclerosis and CVD.

We found dialysis vintage was positively associated with serum TMAO independently. Stubbs et al.’s study results also showed a trend across TMAO quintiles, however, without significance statistically [28]. Another study did not found the relation between dialysis vintage and TMAO [6]. It needs further prospective studies to clarify. In our opinion, longer dialysis vintage, more altered gut microbiota and higher serum TMAO level. As everyone knows, dialysis vintage is also a risk factor for VC in dialysis patients [24].

In addition, higher serum urea levels were associated with higher TMAO concentrations independently in our cross-sectional study. It was consistent with the results of Shafi et al.’s study which utilized the data from the Hemodialysis (HEMO) Study, a United States multicenter trial of dialysis dose and flux [6]. Similarly, Stubbs et al.’s study in a subset of the EVOLVE trial also found the higher TMAO quintiles, the higher serum urea concentrations [28]. The relation between TMAO and urea may be explained by the various diets. One of the main precursors of TMA is l-carnitine, an amino acid in red meat, which is also the dietary source for urea. Alternatively, Meersman et al. demonstrated that TMAO could protect living organisms from the protein denaturing effects originated from high levels of urea [29]. Based on these, we can speculate that more protein intake or increased protein catabolism due to dysbiosis of gut microbiota was related to higher urea and TMAO. The processes also meant higher phosphate intake or the inflammation state in HD patients, both of which will accelerate VC [24]. Urea effects on the intestinal epithelium, vascular wall can promote systemic inflammation, VC [30].

Several studies have shown a positive association between the serum concentration of TMAO and inflammation biomarkers, such as usCRP, IL-1β, and so on, in general population or patients with stable angina [31,32]. However, we found no association between TMAO and usCRP in 184 HD patients, consistent with Wilson Tang et al.’s study in 521 CKD patients [17]. In contrast, Missailidis et al.’s study observed a weak positive correlation between TMAO and usCRP, which was disappeared when GFR was taken into account in non-HD CKD patients [7], whereas Kaysen et al. reported a paradoxical inverse association between TMAO and usCRP in HD patients [33]. Though we cannot explain the different outcomes in these studies, it may be related to many confounding factors, such as different inflammation state in those groups, different GFRs, with or without HD patients, relatively small sample sizes and so on. Therefore, the relationship between TMAO and usCRP in CKD patients needs to be further determined in larger samples. It is worth noting that *in vitro* studies performed in cultured endothelial progenitor cells (EPCs) showed that TMAO promoted cellular inflammation and increased oxidative stress [32].

Our study had several limitations. This was a cross-sectional observational study; thus it cannot provide a causal relationship to the results of the study. The sample size of the study was relatively small and it was a single center study. There were still some confounding factors such as diets, drugs, and so on that may affect the results. Controls were not matched well and AAC score was not measured in control group. TMAO was measured only once and changes in diet and gut microbiome over time may change serum TMAO concentrations.

## Conclusions

Our study provided the clinical evidence for the first time that TMAO, the gut microbiota-derived metabolite, was an independent related factor of VC in HD patients. Higher serum TMAO, older age, longer dialysis vintage, higher plasma iPTH and with diabetes mellitus were related to increased AAC in HD patients independently. Serum TMAO were associated with glucose and lipid metabolism in HD group. The underlying mechanism about TMAO and VC is worth to be further explored. These findings may hint at a new therapeutic pathway for VC.

## Data Availability

All the data generated or analyzed during this study are included in this article. Further enquiries can be directed to the corresponding author.
